# Long-term macrolide treatment for non-cystic fibrosis bronchiectasis in children: a meta-analysis

**DOI:** 10.1038/s41598-021-03778-8

**Published:** 2021-12-20

**Authors:** Eun Lee, In Suk Sol, Jong Deok Kim, Hyeon-Jong Yang, Taek Ki Min, Gwang Cheon Jang, Yoon Ha Hwang, Hyun-Ju Cho, Dong In Suh, Kyunghoon Kim, Hwan Soo Kim, Yoon Hee Kim, Sung Il Woo, Yong Ju Lee, Sungsu Jung, You Hoon Jeon

**Affiliations:** 1 Department of Pediatrics, Chonnam National University Hospital, Chonnam National University Medical School, Gwangju, Republic of Korea; 2 Department of Pediatrics, Kangbuk Samsung Hospital, Sungkyunkwan University School of Medicine, Seoul, Republic of Korea; 3 Department of Pediatrics, Severance Hospital, Yonsei University College of Medicine, Seoul, Republic of Korea; 4 Pediatric Allergy and Respiratory Center, Department of Pediatrics, Soonchunhyang University Seoul Hospital, Soonchunhyang University College of Medicine, Seoul, Republic of Korea; 5 Department of Pediatrics, National Health Insurance Service, Ilsan Hospital, Ilsan, Republic of Korea; 6 Department of Pediatrics, Busan St. Mary’s Hospital, Busan, Republic of Korea; 7 Department of Pediatrics, International St. Mary’s Hospital, Catholic Kwandong University, Incheon, Republic of Korea; 8 Department of Pediatrics, Seoul National University College of Medicine, Seoul, Republic of Korea; 9 Department of Pediatrics, College of Medicine, The Catholic University of Korea, Seoul, Republic of Korea; 10 Department of Pediatrics, Yonsei University College of Medicine, Seoul, Republic of Korea; 11 Department of Pediatrics, Chungbuk National University Hospital, Chungbuk National University College of Medicine, Cheongju, Republic of Korea; 12 Department of Pediatrics, Yongin Severance Hospital, Yonsei University College of Medicine, Yongin, Republic of Korea; 13 Department of Pediatrics, Pusan National University Children’s Hospital, Pusan National University School of Medicine, Yangsan, Republic of Korea; 14 Department of Pediatrics, Hallym University Dongtan Sacred Heart Hospital, 7, Keunjaebong-gil, Hwaseong-si, Gyeonggi-do 18450 Republic of Korea

**Keywords:** Diseases, Medical research

## Abstract

Recurrent bacterial infection causes frequent bronchiectasis (BE) exacerbations. The effectiveness and safety of long-term administration of macrolides in BE remain controversial, especially in children who require minimal treatment to prevent exacerbation. We conducted this meta-analysis to determine the usefulness of long-term macrolide use in pediatric BE. We searched PubMed, Cochrane Library databases, Embase, KoreaMed, Igaku Chuo Zasshi, and Chinese National Knowledge Infrastructure databases. We identified randomized controlled trials (RCTs) which elucidated long-term macrolide treatment (≥ 4 weeks) in non-cystic fibrosis BE in children aged < 18 years. The primary outcome was frequency of acute exacerbation; secondary outcomes included changes in pulmonary function, sputum scores, and adverse events including bacterial resistance. We included four RCTs. Long-term macrolide treatment showed a significant decrease in the frequency of exacerbation (odds ratio [OR], 0.30; 95% confidence interval [CI], 0.10–0.87), mean number of exacerbations per patient (mean difference, − 1.40; 95% CI, − 2.26 to − 0.54), and sputum purulence score (mean difference, − 0.78; 95% CI, − 1.32 to − 0.24). However, long-term macrolide treatment was accompanied by an increased carriage of azithromycin-resistant bacteria (OR, 7.13). Long-term macrolide administration prevents exacerbation of BE in children; however, there are risks of increasing antibiotic resistance. Benefits and risks should be weighed and determined on a patient-by-patient basis.

## Introduction

Bronchiectasis (BE) is a chronic lung disease characterized by an irreversible dilatation and distortion of the small airways including cartilage accompanied by a hyperconcentrated airway mucus^1,2^. Diverse diseases associated with respiratory infections can cause non-cystic fibrosis BE in children^3^. With an increasing awareness of the disease and accessibility of medical resources, BE is no longer considered an orphan disease as the prevalence of BE is increasing^4^. BE is a burdensome condition because it is accompanied by frequent exacerbations^3^. The quality of life of patients is significantly reduced due to repeated hospitalizations secondary to frequent exacerbations, especially in children, which also affects the quality of life of the entire family. Families with BE pediatric patients may experience school/work absences; therefore, this affliction is significant in terms of the disease burden^5^.

Perturbation and alteration of bacteria in the airway of patients with BE is associated with persistent airway inflammation, chronic purulent sputum production, and recurrent lower respiratory infection^6^. Frequent exacerbations of BE, which is closely linked with the deterioration of pulmonary function, poor quality of life, and reduced lifespan, play a critical role in the progression of BE with chronic persistent airway inflammation. Therefore, the prevention of exacerbation in patients with BE is necessary to stabilize airway inflammation, especially in children, when considering its influence over a lifetime.

Besides the antibiotic effects, macrolides have immunomodulatory effects through the regulation of pro-inflammatory and anti-inflammatory immune responses^7^. Based on this evidence^7^, long-term macrolide treatment has been administered to control BE exacerbations. Although several meta-analyses on the effects of long-term macrolide treatment for the prevention of exacerbation and improvement of lung function and quality of life have been performed in cystic fibrosis and non-cystic fibrosis BE in adult patients^8^, data on pediatric non-cystic fibrosis BE are lacking. The present meta-analysis was performed to determine the efficacy and safety of long-term macrolide treatment for non-cystic fibrosis BE in children.

## Materials and methods

### Literature search

The following databases were searched: PubMed, Cochrane Library databases, Embase, KoreaMed, Igaku Chuo Zasshi (ICHUSHI), and Chinese National Knowledge Infrastructure (CNKI) on July 2, 2020. There were no language restrictions. Search terms included “Macrolides,” “Azithromycin,” “Clarithromycin,” “Roxithromycin,” “Erythromycin,” “Bronchiectasis,” “Kartagener syndrome,” and “Ciliary motility disorders”.

### Inclusion criteria

Randomized controlled trials (RCTs) of more than 4 weeks of macrolide treatment that compared with placebo or no intervention for long-term management of stable BE in infants, children, and adolescents under the age of 18 years were included in the present meta-analysis. Ciliary motility disorders, including Kartagener syndrome, were included.

### Exclusion criteria

We excluded RCTs conducted in BE with cystic fibrosis in children and adult patients. Studies performed for insufficient periods were also excluded.

### Primary and secondary outcomes

The primary outcomes were exacerbations of BE. The BE exacerbations were assessed as frequency of exacerbations and hospitalization due to exacerbations. The secondary outcomes were changes in pulmonary function, including forced expiratory volume in 1 s (FEV_1_) and forced vital capacity (FVC), sputum scores, cytokines in sputum/bronchoalveolar lavage (BAL) fluid, and adverse events including bacterial resistance.

### Data extraction and analysis

All references were independently extracted by two reviewers. De-duplicated studies were imported into Covidence online software (https://www.covidence.org). Two review authors reviewed the titles and abstracts of de-duplicated studies and chose the relevant studies. Any discrepancies were solved through discussion. Then, two reviewers independently reviewed the full text of the selected article once again. We performed data extraction including participants’ data, inclusion/exclusion criteria, intervention details, and outcome measurements. Any differences in the data extraction were resolved by discussion and, if necessary, consultation with a third reviewer.

### Assessment of quality and the level of evidence

Quality was assessed by two reviewers independently using the criteria outlined in the Cochrane Handbook for Systematic Reviews of Interventions^9^ according to the seven domains: random sequence generation, blinding of patients, allocation concealment, selective reporting, incomplete outcome data, and other biases. Each of these domains was rated as low, high, or unclear risk. The level of evidence was assessed with the GRADE approach (GRADE pro, Version 3.6 for Windows, Grade Working group)^10^.

### Statistical analysis

The final selected RCTs were combined using Review Manager 5.2.5 (Copenhagen: The Nordic Cochrane Centre, The Cochrane Collaboration, 2012). The heterogeneity of RCTs was accessed using Cochrane Q statistic. After evaluating for heterogeneity of RCTs with *I*^2^ statistic, a random effects model was applied. Odds ratio (OR) for dichotomous variables and mean differences for continuous variables with 95% confidence intervals (95% CI) were calculated. *P* < 0.05 was considered statistically significant.

## Results

### Literature review and selection

**Figure 1:**
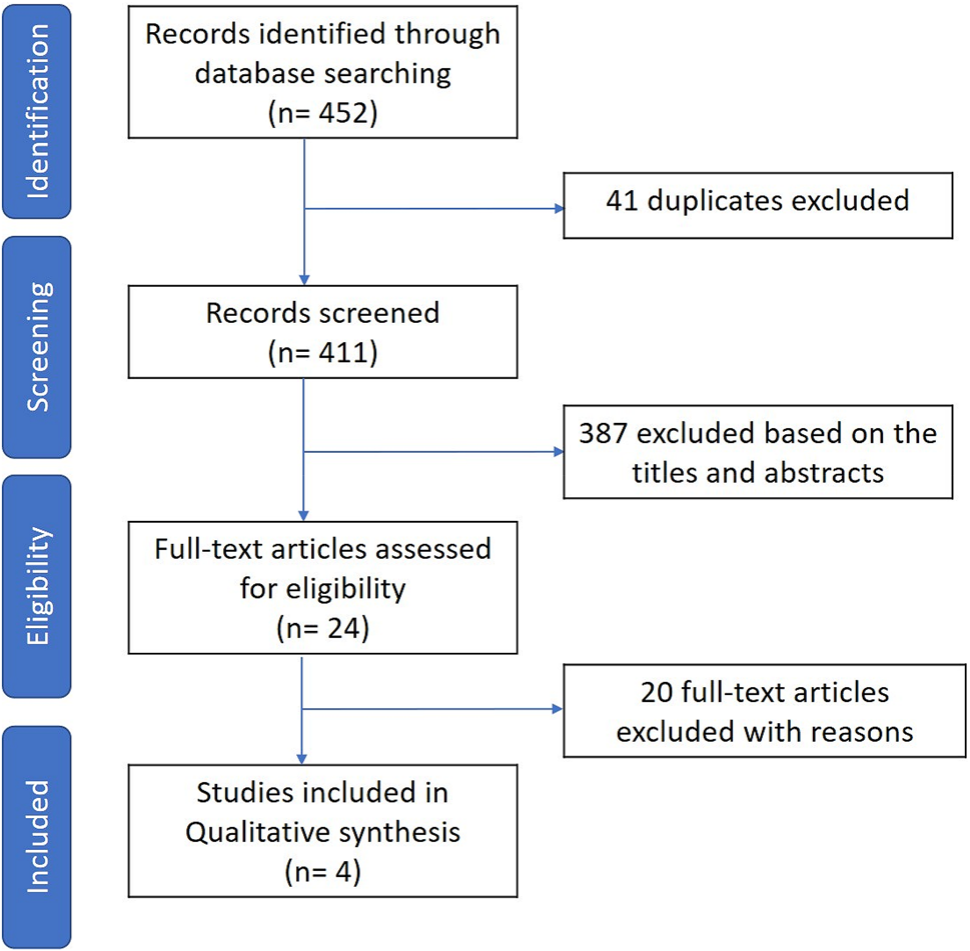
PRISMA flow diagram.

We searched 452 articles, of which 41 were excluded because of duplication. After screening the titles and abstracts, 387 records were removed due to irrelevant publishing types or studies. Four of the 24 full-text articles were qualified for analysis. Figure 1 describes the Preferred Reporting Items for Systematic Reviews and Meta-Analyses (PRISMA) of this meta-analysis.

### Characteristics of the included RCTs

Four eligible RCTs were included in the present meta-analysis. The summarized characteristics of the included RCTs are shown in Table 1. There were a total of four studies administering roxithromycin^11^, erythromycin^12^, azithromycin^13^, and clarithromycin^14^, respectively. The macrolide formulations used in each study differed in both their composition and dosage. In the roxithromycin study^11^, 13 children aged 10–18 (mean age, 13.3 years) were administered 4 mg/kg, twice a day for 12 weeks^11^. In the erythromycin study, 17 children with a mean age of 9.1 were administered 125 mg (less than 15 kg) or 250 mg (more than 15 kg) once a day for a total of 52 weeks^12^. In the azithromycin study, 45 children aged 1 to 8 years (mean age, 4.0 years) were administered 30 mg/kg, once a week, for 24 months^13^. The clarithromycin study administered 15 mg/kg of clarithromycin once a day for three months in 17 children, along with the use of mucolytics and chest physiotherapy for 17 children (mean age, 13.1 years)^14^.

**Table 1:** Summary of the randomized controlled trials on long-term macrolide administration in children with non-cystic fibrosis bronchiectasis.

Study	Characteristics				Intervention and study duration		Outcome
	Country	Year	Number of subjects; mean age of experiment group, y	Number of subjects; mean age of control group, y	Experimental group	Control group	
Koh, 1997	South Korea	1995–1996	13; 13.3 ± 2.5	12; 12.9 ± 2.6	Roxithromycin 4 mg/kg twice a day for 12 weeks	Placebo for 12 weeks	FEV1, PD20, exacerbation, sputum purulence score, sputum leukocyte score
Masekela, 2013	South Africa	2009–2011	17; 8.4 ± 2.4	14; 9.1 ± 2.1	Erythromycin 125 mg (≤ 15 kg), 250 mg (> 15 kg) once a day for 52 weeks	Placebo group for 52 weeks	Number of exacerbations, PFT (FEV1, FVC), cytokines
Valery, 2013	Australia	2008–2010	45; 3.99 ± 2.14	44; 4.22 ± 2.3	Azithromycin (30 mg/kg) once a week for up to 24 months	Placebo once a week for up to 24 months	Exacerbation rate (respiratory episodes treated with antibiotics)
Yalcin, 2006	Turkey	1999–2000	17; 13.1 ± 2.7	17; 11.9 ± 2.9	Clarithromycin 15 mg/kg, once daily with supportive therapies (mucolytic and expectorant medications, postural drainage) for 3 months	Supportive therapies (mucolytic and expectorant medications, postural drainage) for 3 months	Sputum production, PFT (FEF 25–75%), cytokines and culture using BAL fluids

BAL, Bronchoalveolar lavage; FEF 25–75%, forced expiratory flow at 25–75% of forced vital capacity; FEV1, forced expiratory volume in 1 s; FVC, forced vital capacity; PD20, provocative dose of methacholine causing a 20% fall in FEV1; PFT, pulmonary function test.

### Outcomes

#### Primary outcomes: exacerbations of bronchiectasis

**Figure 2:**
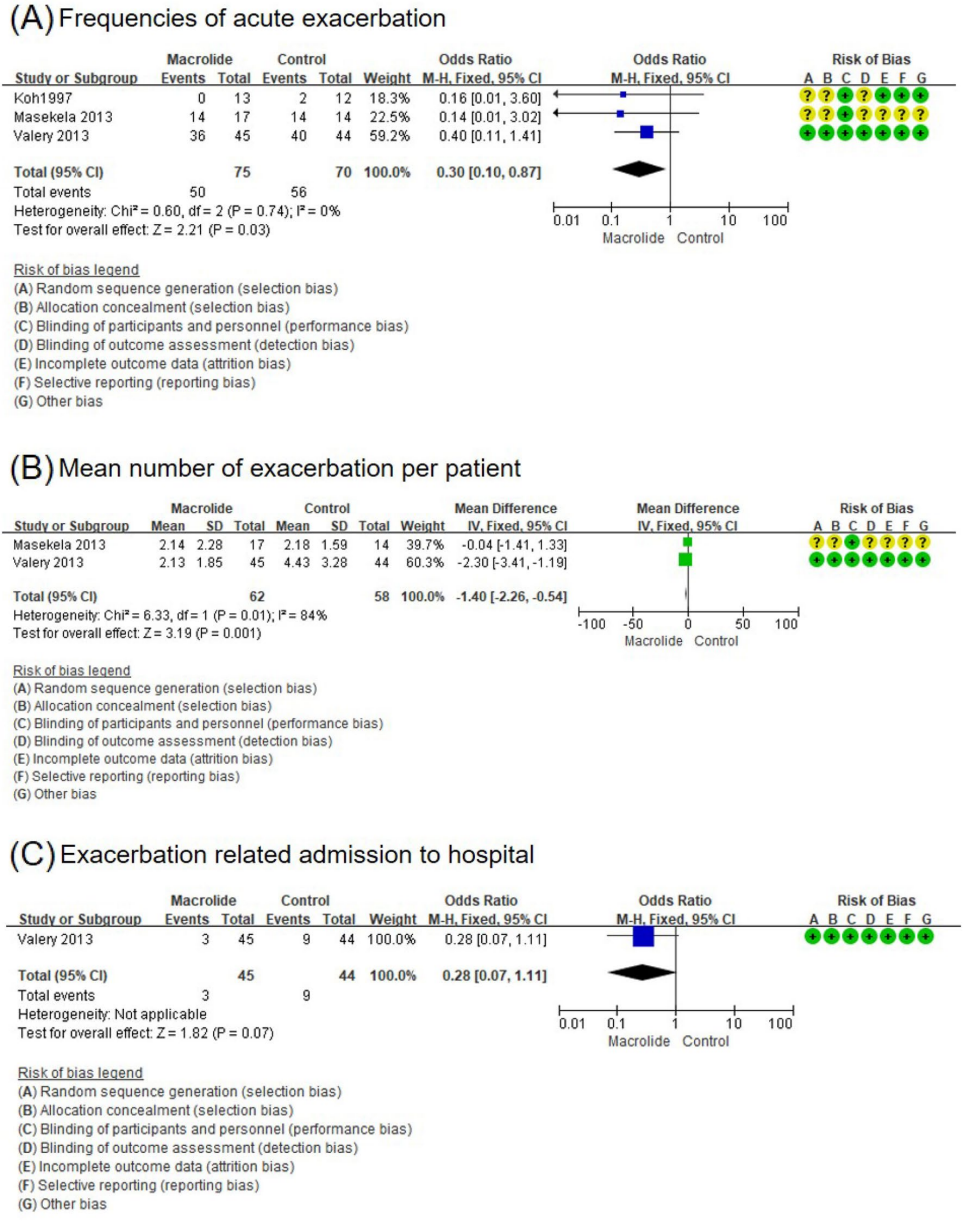
Forest plot of the effects of long-term macrolide treatment on acute exacerbation of bronchiectasis (BE) in children with non-cystic fibrosis BE. (**A**) Frequencies of acute exacerbation, (**B**) mean number of exacerbations of BE per patient, and (**C**) exacerbation-related admission to the hospital.

Three trials investigated the effects of long-term macrolide treatment on the frequency of acute exacerbations of BE^11–13^. Although macrolide preparations were different, long-term use of macrolides significantly reduced the frequency of acute exacerbations of BE (OR, 0.30; 95% CI, 0.10 to 0.87) (Fig. 2A). With long-term macrolide treatment, the mean exacerbations of BE per patient was also significantly reduced (mean difference, − 1.40; 95% CI, − 2.26 to − 0.54) (Fig. 2B)^12,13^. One study was available to examine the effect of long-term azithromycin treatment on hospitalization due to exacerbations of BE. There was no significant difference in the frequency of exacerbation-related admissions in the azithromycin group compared to the control group (OR, 0.28; 95% CI, 0.07–1.11) (Fig. 2C)^13^.

#### Secondary outcome: pulmonary function

**Figure 3:**
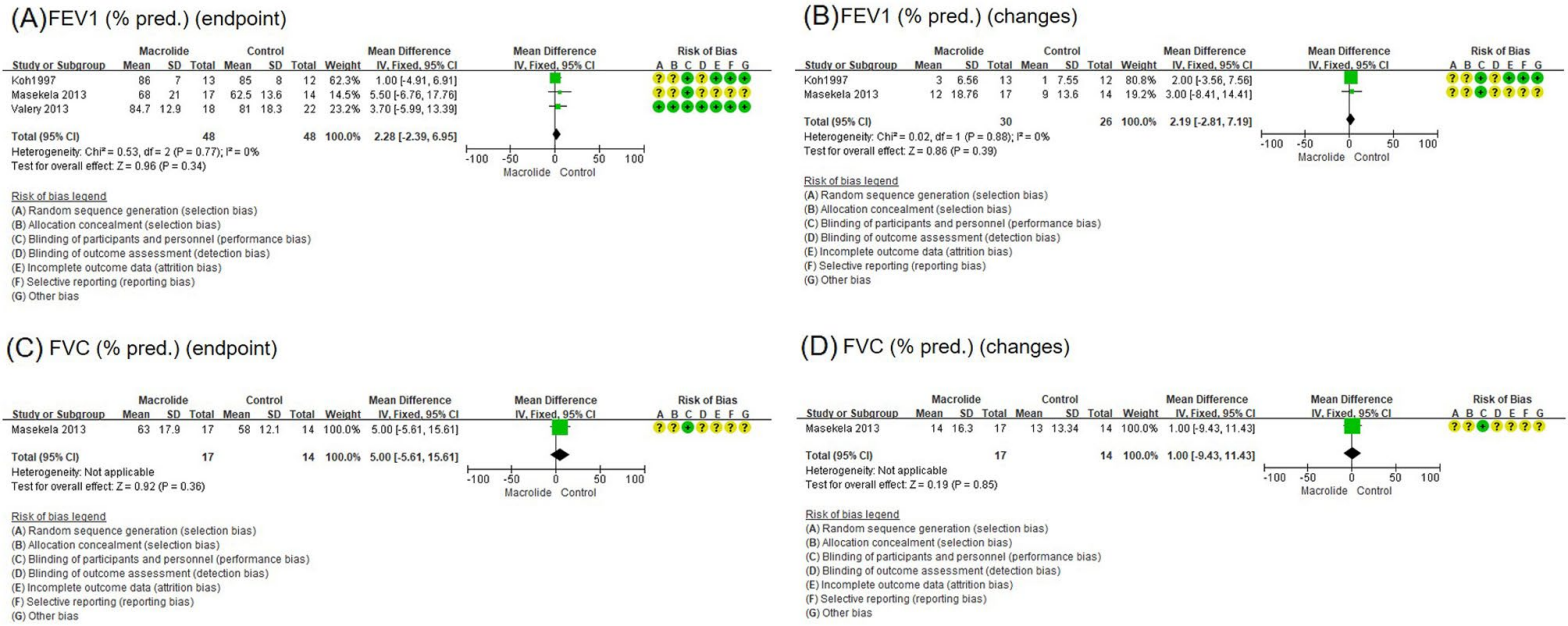
Forest plot of the effects of long-term macrolide treatment on the pulmonary function in children with non-cystic fibrosis BE. (**A**) FEV1% predicted at endpoint, (**B**) FEV1% predicted changes, (**C**) FVC % predicted at the endpoint, and (**D**) FVC % predicted changes. FEV1, forced expiratory volume in 1 s; FVC, forced vital capacity.

There were three studies on the effect of macrolide long-term treatment on FEV1% predicted at endpoint in children with BE^11–13^. There was no significant difference between FEV1 after treatment of a macrolide and control (mean difference, 2.28; 95% CI, − 2.39 to 6.95) (Fig. 3A).

Two studies were conducted on the effect of long-term macrolide treatment on changes in the FEV1% predicted in patients with BE^11,12^. There was no significant difference in changes of FEV1% predicted before and after treatment in the macrolide group and control (mean difference, 2.19; 95% CI, − 2.81 to 7.19) (Fig. 3B).

One trial studied the effect of long-term erythromycin treatment on FVC at endpoint in children with BE^8^. In the macrolide-treated group and the control group, the FVC at endpoint had no significant difference (mean difference, 5.00; 95% CI, − 5.61 to 15.61) (Fig. 3C). One study published on the effects of long-term erythromycin treatment on changes in the FVC% predicted in patients with BE^12^. There was no significant difference in the FVC changes after long-term macrolide treatment from controls (mean difference, 1.00; 95% CI, − 9.43 to 11.43) (Fig. 3D).

#### Secondary outcome: sputum score

**Figure 4:**
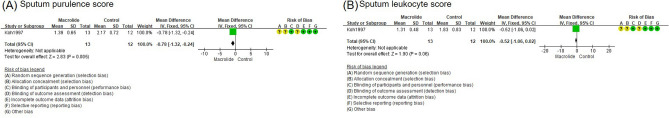
Forest plot of the sputum scores of long-term macrolide treatment on children with non-cystic fibrosis BE. (**A**) Sputum purulent score, and (**B**) sputum leukocyte score in children with BE.

Data on the effect of long-term roxithromycin treatment on sputum production were available in one trial^11^. The difference between the macrolide-treated group and the comparator's sputum purulence score was − 0.78 (95% CI, − 1.32 to − 0.24), meaningfully decreasing in the macrolide-treated group (Fig. 4A). There was no significant decrease in the sputum leukocyte score in the macrolide-treated group compared to the control group (Fig. 4B)^7^.

#### Secondary outcome: cytokines

**Figure 5:**
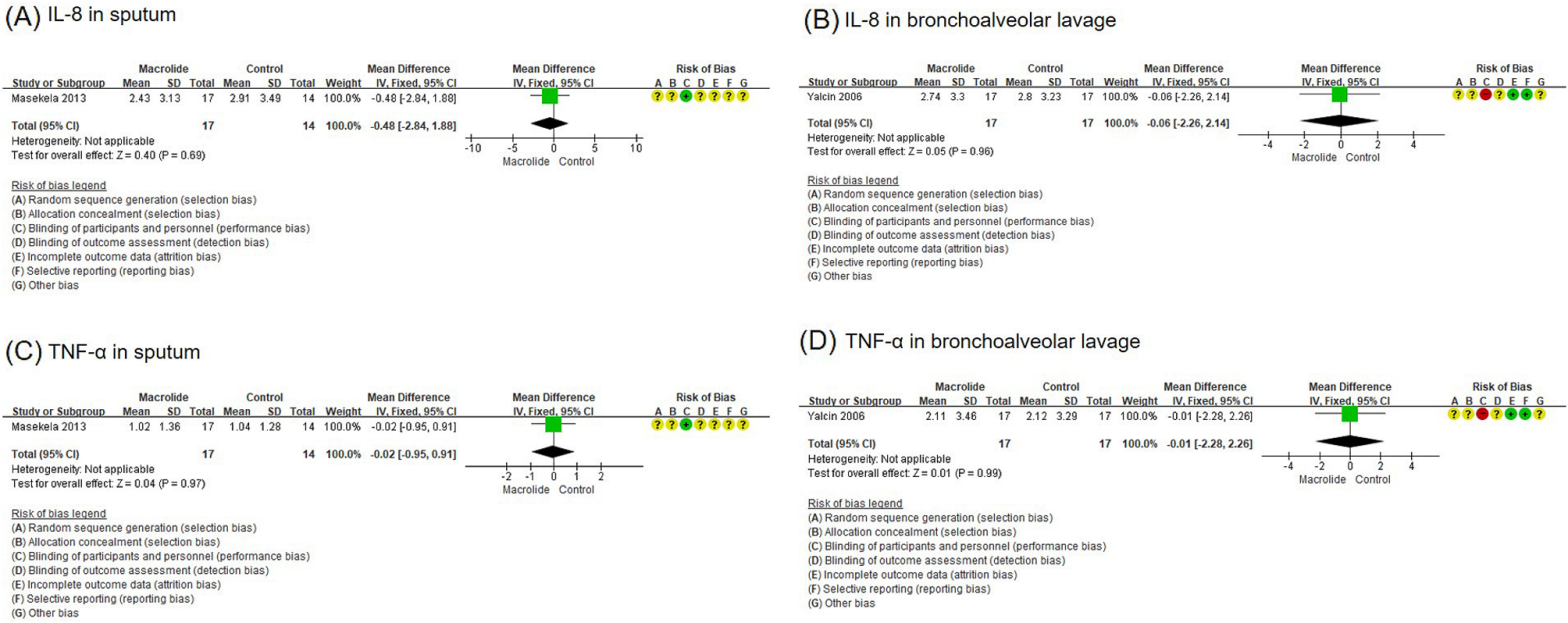
Forest plot of the effects of long-term macrolide treatment on cytokine levels from the sputum in children with BE. (**A**) Log-transformed IL-8 levels in the sputum, (**B**) log-transformed IL-8 levels in the bronchoalveolar lavage fluid, (**C**) log-transformed TNF-α level in the sputum, and (**D**) log-transformed TNF-α level in the bronchoalveolar lavage fluid.

One trial investigated the effect of long-term macrolide treatment in the sputum IL-8 levels in children with non-cystic fibrosis BE. The study showed that IL-8 (endpoint, log) levels in the erythromycin-treated group was − 0.48 (− 2.84 to 1.88) and revealed no significant difference from the levels in the control group (Fig. 5A)^9^. There was another study on the effect of long-term macrolide treatment on the IL-8 levels, but the samples were BAL fluid. The study showed that the level of log transformed IL-8 in the BAL fluid after macrolide treatment was − 0.06 (− 2.26 to 2.14), which was not significantly different from the control group (Fig. 5B)^10^.

Another study investigated the effects of erythromycin on the TNF-α level in the sputum^12^. It showed that the difference in the log-transformed TNF-α levels after macrolide treatment in the macrolide-treated group and control group was − 0.02 (− 0.95 to 0.91), which was not significant (Fig. 5C). There was another study on the effect of long-term macrolide treatment on the TNF-α level in BAL fluid. The study showed no differences in the log-transformed TNF-α levels in the BAL fluid at the end of the study between the macrolide treatment group and control group (mean difference, − 0.01; 95% CI, − 2.28 to 2.26) (Fig. 5D)^12^.

#### Secondary outcome: antibiotic resistance

**Figure 6:**
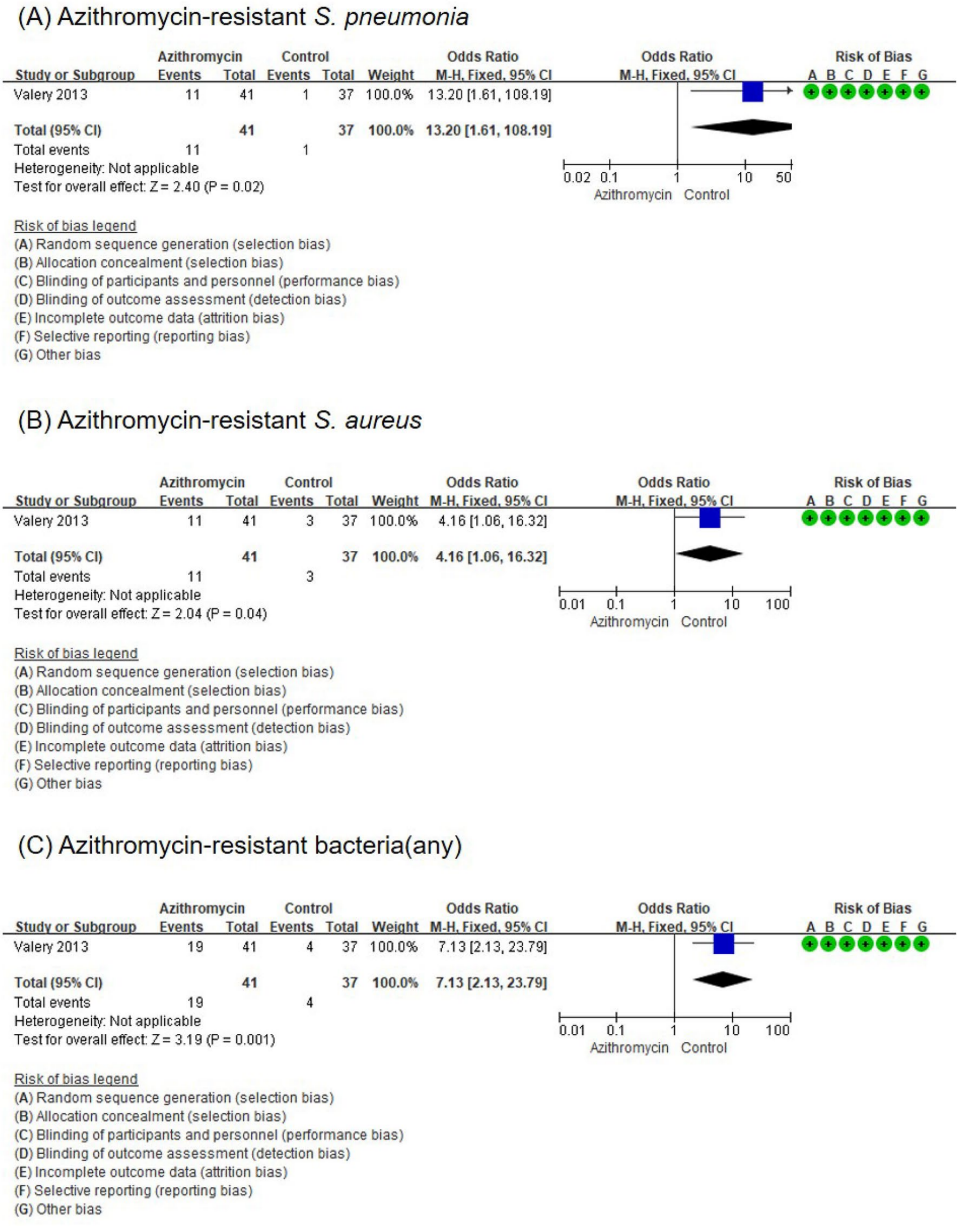
Forest plot of the effects of long-term macrolide treatment on the development of resistance to antibiotics in children with BE. (**A**) Azithromycin-resistant *Streptococcus pneumoniae*, (**B**) azithromycin-resistant *Staphylococcus aureus*, and (**C**) any azithromycin-resistant bacteria.

Data on the emergence of resistance to antibiotics after long-term macrolide therapy was available in one study^13^. The long-term use of azithromycin significantly increased the incidence of the following: azithromycin-resistant *S. pneumoniae* (OR, 13.20; 95% CI, 1.61–108.19) (Fig. 6A), azithromycin-resistant *S. aureus* (OR, 4.16; 95% CI, 1.06–16.32) (Fig. 6B), and azithromycin-resistant bacteria (OR, 7.13; 95% CI, 2.13–23.79) (Fig. 6C).

#### Secondary outcome: other adverse events

**Figure 7:**
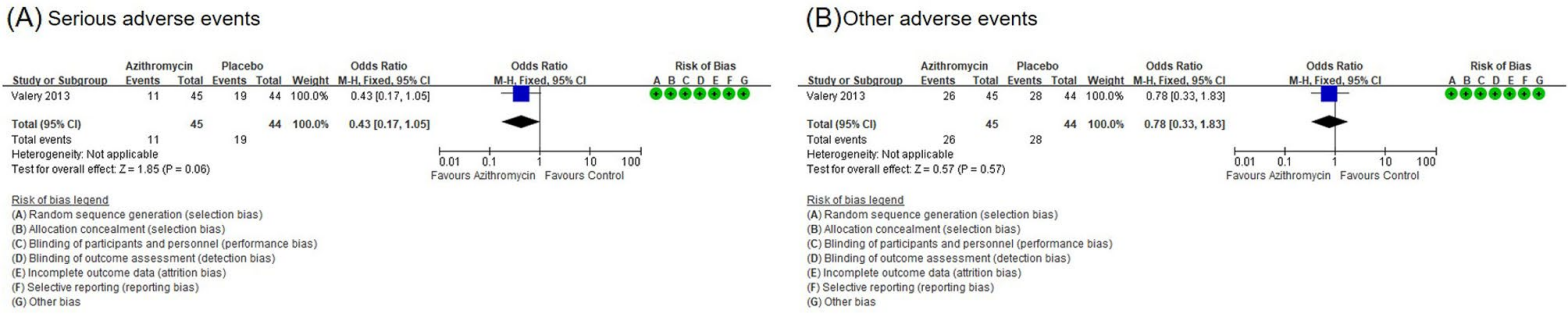
Forest plot for adverse events with long-term macrolide treatment in children with BE. (**A**) Serious adverse events and (**B**) other adverse events.

The macrolide long-term treatment did not increase the incidence of serious adverse events (OR, 0.43; 95% CI, 0.17–1.05) (Fig. 7A)^11^. The macrolide long-term treatment did not increase the incidence of other adverse events (OR, 0.78; 95% CI, 0.33–1.83) (Fig. 7B)^6^.

## Discussion

This meta-analysis shows that long-term macrolide treatment over 3–24 months in pediatric non-cystic fibrosis BE reduces the frequency of exacerbation with a decrease in the mean number of exacerbations per patient during the macrolide treatment period. In addition, we found that long-term macrolide treatment improved the sputum purulence score; however, it did not affect the pulmonary function index and cytokine levels, including IL-8 and TNF-α, in the sputum and BAL fluid. As an adverse effect of long-term macrolide treatment, the rate of azithromycin-resistant bacteria significantly increased (azithromycin-resistant *Streptococcus pneumoniae*; OR: 13.2; azithromycin-resistant *Staphylococcus aureus*; OR: 4.1; any azithromycin-resistant bacteria; OR: 7.13), although no other serious adverse reactions were identified. Our meta-analysis provides an assessment of the advantages and disadvantages of long-term macrolide treatment focused on pediatric non-cystic fibrosis BE.

We meta-analyzed the effects of macrolides for acute exacerbation in pediatric BE with three RCTs that were performed with the administration of roxithromycin for 12 weeks^11^, erythromycin for 52 weeks^12^, and azithromycin for up to 24 months^13^. They showed consistent results on the reduction of the number of exacerbations, although the RCTs were performed for different study durations in different study populations, including immunocompromised children due to HIV infection in one RCT^12^. One study elucidated the effects of clarithromycin on pulmonary function and sputum cytokines, but did not investigate the effect of long-term macrolide treatment on the frequency of BE exacerbations in children^14^. Another showed a significant decrease in sputum purulence score in the roxithromycin group compared to the control group^11^. Although there was heterogeneity in the protocols of the RCTs for macrolides, dosage of macrolides, study duration, age, and characteristics of the study population, we concluded that long-term macrolide treatment has beneficial effects on the reduction of exacerbations and sputum score in children with non-cystic fibrosis BE.

Despite the beneficial effects, it is important to consider the safety of long-term macrolide treatment in children. Among the four RCTs included in the meta-analysis, only one study investigated the adverse reactions^13^. The critical adverse reactions in the long-term macrolide treatment group significantly increased azithromycin-resistant bacteria, including *S. pneumonia* and *S. aureus*, when 30 mg/kg of azithromycin was administered once a week for up to 24 months^13^. In the era of increasing macrolide resistance^15,16^, the increased risk of macrolide resistance in the long-term macrolide treatment in pediatric non-cystic fibrosis BE arouse attention to the widespread use of long-term macrolide treatment in children. The benefits of long-term macrolide treatment and the risks of bacterial resistance must be considered.

Although a previous study reported that administering azithromycin for 5 days can increase the risk of cardiovascular diseases in adults^17^ and long-term macrolide treatment in adults with non-cystic fibrosis BE showed gastrointestinal complications, including diarrhea, nausea, vomiting or abdominal discomfort^18^, no adverse events were reported in long-term macrolide treatment in cases of pediatric non-cystic fibrosis BE compared to the control group^13^. In addition, there was no reported severe adverse events of long-term macrolide treatment in children with non-cystic fibrosis BE. To identify and prevent the adverse reactions of long-term macrolide treatment, regular monitoring of the possible complications, such as cardiovascular diseases, is warranted in future studies. Despite these adverse reactions, long-term macrolide treatment can be one of the treatment options that can decrease the exacerbation of non-cystic fibrosis BE in children, if other treatment strategies are insufficient.

There are some limitations to the present study. The RCTs in the present meta-analysis are different in terms of the characteristics of the study population (age, sex, and severity of BE, and immune status), study duration, and macrolide classes. Despite the protocols’ heterogenicity, this meta-analysis analyzed limited results, and it is clear that long-term macrolide treatment helps prevent pediatric BE from deteriorating. However, the question remains whether to choose between the benefits of preventing further progression and the risk of increased antibiotic resistance. The findings of this meta-analysis can be one of the alternatives for the long-term management of pediatric non-cystic fibrosis BE in the absence of a definite treatment other than the administration of antibiotics during exacerbation^1^.

In conclusion, long-term macrolide treatment prevents exacerbation of BE in children, but increases antibiotic resistance. Long-term macrolide treatment should be individualized and cannot be administered in all pediatric BE patients due to its accompanying risks. However, long-term macrolide therapy is still one of the treatment options, considering the unavailability of other special treatments for BE exacerbation prevention and the poor quality of life for pediatric patients. Still, the decision to treat should be made in consideration of various factors, such as the condition of the individual patients, duration of disease, and quality of life. Valuable studies on the treatment of pediatric BE are warranted.
